# 723. Clinical and demographic features associated with isolation of ESBL-producing *Enterobacteriaceae* in a private hospital in Kingston, Jamaica

**DOI:** 10.1093/ofid/ofad500.784

**Published:** 2023-11-27

**Authors:** Eugene W Liu, Matthew Mendoza, Hansel Fletcher, Fredricka Coombs

**Affiliations:** Loma Linda University, Loma Linda, California; Loma Linda University, Loma Linda, California; Loma Linda University, Loma Linda, California; Andrews Memorial Hospital, Kingston, Kingston, Jamaica

## Abstract

**Background:**

Emergence of antibiotic resistant bacteria is a public health threat. Data on the burden of ESBL-producing bacteria is limited, particularly in low to middle income countries, such as Jamaica. Here we sought to identify clinical and demographic features associated with extended spectrum β-lactamase producing Enterobacterales (ESBLs) in patients at Andrews Memorial Hospital (AMH), a private hospital in Kingston, Jamaica.

**Methods:**

This retrospective case control study compared patients with cultures with and without ESBLs. Cases and controls were identified from 2021 microbiology records. Case subjects were AMH patients with ≥ 1 Enterobacterales isolate resistant to ceftriaxone, and controls were individuals without any ESBLs isolated on culture in 2021 with at least one culture (including ones without growth) performed the first week each month. We included all possible cases and performed stratified random sampling by month of controls to reach a 1:2 case:control ratio. We identified cultures of interest, defined as the first culture with an ESBL isolate for each case and the first culture from the random sample for each control. For each culture of interest, we extracted electronic medical record data including antibiotics received in the preceding 6 months, organism, sex, and age.

**Results:**

Among 35 cases and 48 controls, age (1.04; 95%CI 1.01-1.06); male sex (5.46; 1.93-15.50); and isolation of *Enterobacter* species (10.93; 1.23-97.04) were associated with increased odds of ESBL isolation on simple logistic regression. Bidirectional stepwise variable selection indicated these associated factors should be included in a multiple logistic regression model. In this model, age (1.05; 1.00-1.09) and isolation of *Enterobacter* species (17.53; 1.39-220.76) were independently associated with increased odds of ESBL isolation.

Table 1

**Figure:**
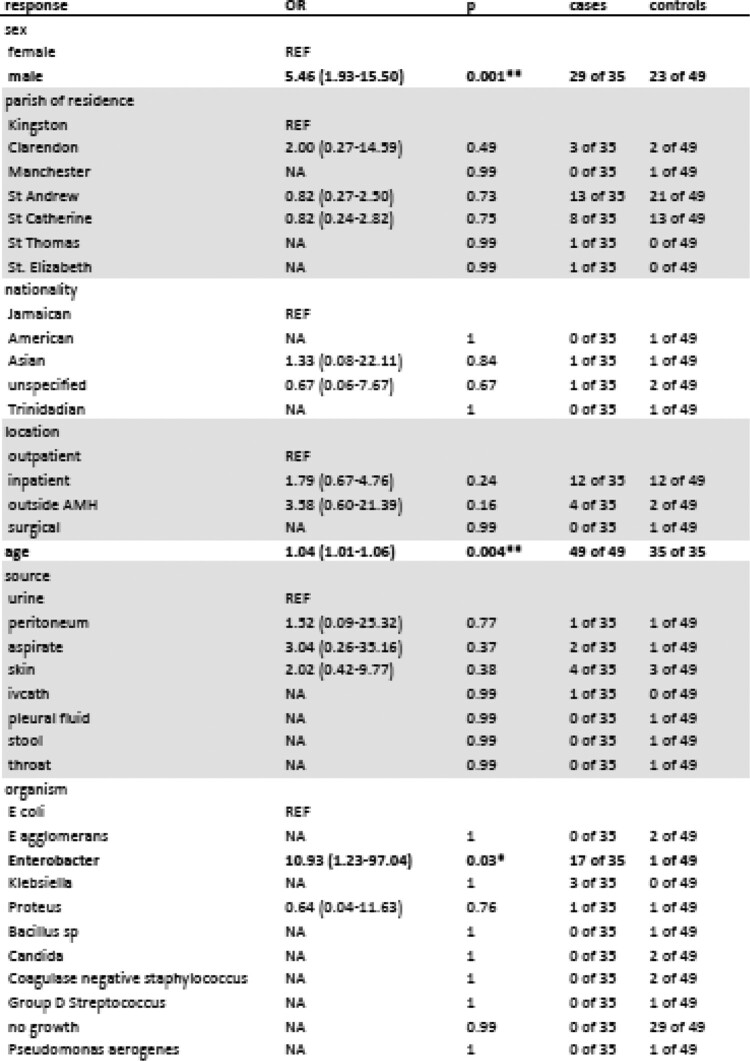

Risk Factors for Isolation of ESBL in AMH Patients, as Estimated with Simple Logistic Regression Models—2021​

Table 2

**Figure:**
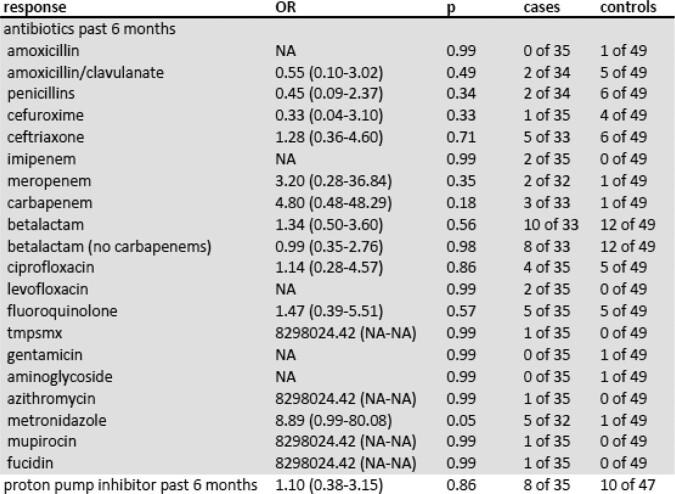

Medication Risk Factors for Isolation of ESBL in AMH Patients, as Estimated with Simple Logistic Regression Models—2021​

**Conclusion:**

Age was independently associated with increased odds of ESBLs and may be a marker for past exposure to β-lactam antibiotics or nosocomial infection with ESBLs. Past exposures may have greater influence on ESBL isolation compared to receipt of antibiotics in the past 6 months where we saw no associations. The association with isolation of *Enterobacter* species and ESBL isolation may be from confounding with ampC inducible resistance.

**Disclosures:**

**All Authors**: No reported disclosures

